# Perceptions of Aging and Control Beliefs: A Study on Older Patients’ Views of Aging

**DOI:** 10.3390/geriatrics10060148

**Published:** 2025-11-10

**Authors:** Aline Schönenberg, Charlotte Kobus, Marlene Günther, Luise Umfermann, Tino Prell

**Affiliations:** 1 Department of Geriatrics, Halle University Hospital, 06120 Halle (Saale), Germany; 2 Department of Geriatrics, Jena University Hospital, 07745 Jena, Germany

**Keywords:** views on aging, locus of control, geriatric, health

## Abstract

Background: Locus of control (LoC) may shape how older adults appraise aging, particularly in acute geriatric rehabilitation. Evidence linking internal/external LoC to domain-specific Views on Aging (VoA, containing Physical Loss, Social Loss, Personal Growth, Self-awareness/Gains) remains limited. Methods: We analyzed a cross-sectional cohort of patients aged 70 and above from an acute geriatric rehabilitation unit (N = 103) and contextualized findings with a 1:1 Mahalanobis-matched subsample from the German Ageing Survey. Internal and external LoC and covariates (age, sex, Barthel, cognitive function, depressive symptoms, health satisfaction) were standardized (z). Associations were estimated using (i) ordinary least squares (OLS) regression across eight LoC effects, as well as (ii) proportional-odds ordinal models (quartiles; logit link), as a complementary, distribution-robust approach. Results: For the Physical VoA domain, higher internal LoC related to more positive appraisals (OLS β = 0.133, 95% CI 0.043–0.223, *p* = 0.035; OR = 3.52), whereas higher external LoC related to less positive appraisals (β = −0.165, 95% CI −0.285 to −0.045, *p* = 0.035; OR = 0.274). Internal LoC also increased the odds of more positive Personal Growth (OR = 1.64, 95% CI 1.04–2.72), while effects on Social Loss (external LoC OR = 0.649, 95% CI 0.418–0.991) and Gains were smaller. Univariate Spearman correlations were directionally consistent. In the DEAS comparison, older patients showed greater endorsement of both physical losses and gains. Conclusions: In acute geriatric rehabilitation, internal control beliefs align with more positive views of physical aging and growth, whereas external control aligns with less positive physical (and modestly social) views. The results position LoC as a clinically relevant correlate of aging appraisals.

## 1. Introduction

The aging process is a complex phenomenon that encompasses a range of physical, psychological, and social changes, which can impact the well-being of older adults [1]. It is of paramount importance to gain an understanding of how older adults perceive advancing age and exert control over their environment in order to fully comprehend their adaptation to the aging process [2]. Theories of aging, such as control theories and self-oriented models, have investigated the influence of perceptions of aging and control beliefs on health, psychological well-being, and adaptation to aging [3]. These discussions are predicated on the notion of views on aging (VoA) [4,5]. VoA play a critical role in shaping health outcomes, well-being, and longevity in older adults [6]. VoA encompass individuals’ perceptions of age, which can be influenced by age stereotypes and self-perceptions of aging [7,8]. These stereotypes are not only pervasive but are often internalized by individuals at an early age, thereby influencing their VoA as they mature [9]. Research indicates that VoA form early in life but become especially impactful during middle and later adulthood. The positive or negative nature of these views can significantly affect mental and physical health, cognitive functioning, and social integration [2,6]. Empirical studies have further demonstrated that positive views on aging contribute to resilience, whereas negative stereotypes can exacerbate feelings of helplessness and reduce engagement in health-promoting behaviors [6].

Locus of control (LoC), a construct developed by Rotter within the framework of social learning theory, refers to the extent to which individuals perceive their actions as influencing outcomes in life. An internal locus of control is characterized by the conviction that one’s actions are the primary determinant of life events. This internal LoC appears beneficial for many indicators of health and well-being [10]. In contrast, an external LoC is typified by the perception that external forces, such as fate or the influence of powerful others, are the primary drivers of outcomes. This belief can be associated with negative behavioral, affective, and functional outcomes, including poorer mental and physical well-being [10]. Control theories, such as Heckhausen and Schulz’s Life-Span Theory of Control and Brandstädter and Greve’s Model of the Aging Self, similarly emphasize the significance of control beliefs in the process of aging [11,12]. In accordance with these theories, older adults undergo a transition from a primary LoC, whereby external events are influenced, to a secondary LoC, whereby internal states and expectations are adjusted, as they age.

In the context of aging, both VoA and LoC play a pivotal role in how older adults navigate the challenges they encounter. Negative age stereotypes have been demonstrated to contribute to an external LoC, which in turn fosters feelings of helplessness and diminishes psychological well-being [13]. Conversely, positive perceptions of aging have been shown to reinforce an internal LoC, enabling older adults to maintain a sense of agency and adaptability [3]. Therefore, the combination of positive aging perceptions and an internal LoC is associated with enhanced health outcomes, psychological well-being, and successful adaptation to the aging process.

This relationship between VoA and LoC is particularly relevant in older adults, where declines in physical health and changes in social roles can amplify the need for effective coping strategies. A comprehensive understanding of how older adults adapt to aging, maintain psychological well-being, and engage in health-promoting behaviors requires the study of both constructs together. This research presents empirical data for this cohort and aims to inform interventions designed to improve quality of life by promoting positive VoA and fostering an internal LoC in older adults.

## 2. Materials and Methods

### 2.1. Study Design

#### 2.1.1. ZASSA Cohort

This cross-sectional observational study was conducted between July 2023 and January 2024 in the Centre for Geriatrics in Southern Saxony-Anhalt (Zentrum für Altersmedizin im südlichen Sachsen-Anhalt, ZASSA). We included patients aged 70 years or older with multimorbidity receiving geriatric early rehabilitative complex treatment, a specialized hospital treatment approach for older adults with acute illnesses or injuries, recorded under the Operations and Procedures Key (OPS) system 8-550 [14]. Patients who were not able to provide valid self-report due to severe health problems, delirium or severe dementia were excluded. All patients gave written informed consent. The local ethics committee of Halle University Hospital approved the study (No. 2022-026).

#### 2.1.2. DEAS Cohort

In addition, we extracted data for VoA from the DEAS (Wave 2021) to compare our hospital-based study sample with a representative population-based sample. The DEAS is a nationwide representative cross-sectional and longitudinal survey of German adults aged over 40. It is funded by the Federal Ministry for Family Affairs, Senior Citizens, Women and Youth (BMFSFJ). The comprehensive surveying provides micro-data for social and behavioral scientific research and for the reporting on social developments.

### 2.2. Variables of Interest

To assess VoA, both the ZASSA cohort and the DEAS used the “Bereichsspezifisches subjektives Alterserleben” (Area-specific subjective experience of old age). It covers views on age-related changes in four distinct subscales based on the respective sum scores: Physical Loss (refers to the view of aging as accompanied by physical losses), Social Loss (e.g., no longer being needed by others or decreased respect), Personal Growth (implies that aging is also seen as a time of ongoing personal development), and Self-awareness/Gains. The participants rated the items on a scale ranging from 1 (definitely true) to 4 (definitely false [6]. Note on direction: On the original VoA response format (1 = definitely true–4 = definitely false), lower means indicate stronger agreement. For modeling and between-cohort comparisons we therefore use harmonized domain scores so that higher values consistently indicate more positive VoA.

We used the German Internal–External LoC Short Scale–4 (IE-4) scale to measure internal and external control-beliefs (internal and external LoC). The scale consists of four items, two each for internal and external control-beliefs. Participants responded on a 5-point Likert scale reaching from 1 “strongly disagree” to 5 “strongly agree”, which are each added to the respective sum score [15].

Health satisfaction (HS) was assessed using item G4 from the German version of the WHO Quality of Life questionnaire (WHOQOL), which asks: “How satisfied are you with your health?” Responses were rated on a 5-point Likert scale ranging from 1 (“very dissatisfied”) to 5 (“very satisfied”).

### 2.3. Covariates

To describe the cohort, we collected sociodemographic and medical parameters:

- Age (years, metric), sex (male/female, dichotomous);
- Marital state (single/married/widowed/separated, multinominal);
- Living situation (alone/with partner or family/assisted living/nursing home/others, multinominal);
- Barthel Index [16,17], describes functional level for ten daily tasks (including eating, personal hygiene, mobility, continence), with higher values indicating greater independence;
- Cognitive function measured by Mini-Mental State Examination (MMSE) screens orientation, memory, attention, calculation, and language ability with a maximum of 30 points [18], and higher scores indicate better cognitive function;
- Geriatric depression screening scale (GDS): 15 items assessing emotion and behavior towards life [19], with higher scores indicating more depressive symptoms.

### 2.4. Statistical Analyses

All calculations were performed using SPSS (28.0) and R (4.1.1) using the packages sandwich, lmtest, broom, car, ordinal, dplyr, haven, and tibble. The cohort was characterized using descriptive statistics. The Shapiro–Wilk test was employed to test for normal distribution. The significance level was set at *p* = 0.05.

The study utilized a matched cohort design to compare our ZASSA cohort with the DEAS cohort. To ensure comparability between the two cohorts, a Mahalanobis distance matching approach was applied using the MatchIt package in R. Individuals from the DEAS dataset were selected based on their similarity to participants from our cohort in terms of age, gender, and number of medications (as an indicator for multimorbidity). Matching was conducted using the nearest neighbor algorithm with a 1:1 ratio, minimizing baseline differences between the groups. Individuals from DEAS who best matched the patients from our cohort were included in the final analysis, while non-matched cases were retained for sensitivity analyses.

All VoA items used four response options (1 = “trifft genau zu”, 2 = “trifft eher zu”, 3 = “trifft eher nicht zu”, 4 = “trifft gar nicht zu”; lower values indicate stronger agreement). Domain scores were computed as means of their items. Because the *loss* domains are negatively worded, whereas the resources domains are positively worded, we harmonized the direction so that higher scores consistently reflect a more positive view of aging in every domain. Specifically, the loss domains were kept in their original direction—Physical Loss and Social Loss already have higher values, meaning less perceived loss (thus more positive). The resource domains (Personal Growth and Gains) were reversed so that higher values indicate more growth/gains. In the resulting harmonized VoA variables, higher values indicate a more positive VoA.

To allow effect sizes to be interpreted per 1 SD increase, all continuous predictors were z-standardized within the analytic sample (LoC, age, Barthel Index, MMSE, GDS, and health satisfaction). Sex was modeled as a factor (reference = male). Analyses used complete cases.

We quantified associations between LoC and each harmonized VoA domain using two complementary approaches.

1. Linear models with robust standard errors.

For each VoA domain (treated as continuous), we estimated an OLS model with HC3 heteroskedasticity-robust standard errors. We report robust coefficients (β) with 95% CIs and compute partial R^2 for^ the LoC terms using t2/(t2 + df). Multicollinearity was checked via variance-inflation factors (VIF); VIF > 5 would be considered problematic.

2. Ordinal cumulative logit models.

To provide odds-ratio (OR) effect measures without normality assumptions, each harmonized VoA domain was categorized into quartiles (lowest to highest = least to most positive). We fit proportional-odds models (clm, logit link) with the same covariate set as above. ORs are interpreted per 1 SD increase in the predictor as the change in the odds of being in a more positive VoA category. We assessed the proportional-odds assumption using a nominal test. 95% CIs were obtained from profile/Wald intervals as implemented in the software.

Because there are two primary LoC predictors across four VoA domains, we controlled the family-wise testing burden within each model family by applying Benjamini–Hochberg false discovery rate (FDR) correction across the eight LoC effects (4 domains × 2 predictors). Two-sided α = 0.05 was used. As descriptive and sensitivity analyses, we also report Spearman correlations (raw and, where informative, partial Spearman adjusting for the same covariates) to illustrate zero-order patterns relative to adjusted estimates.

## 3. Results

### 3.1. Description of the Cohort

Table 1 provides a summarized description of the older patients. It predominantly consists of individuals living alone, with a significant proportion being widowed or separated. The majority have completed primary or secondary education. A substantial number of individuals require some level of care, either through formal services or family members. HS varies among the cohort: While 14.7% are very dissatisfied with their health and 30.4% are dissatisfied, 35.3% report being satisfied and 6.9% very satisfied.

### 3.2. Locus of Control

For internal LoC, the vast majority (70.9%) of patients believe they have their life fully in their own hands, and 49.5% believe that if they put in effort, they will be successful. In concordance, for external LoC, 68.0% of patients do not feel that their life is largely controlled by others, while 19.4% feel that their plans are often thwarted by fate. Internal and external LoC was not significantly associated with age, sex, or HS (*p* > 0.05). Supplement Table S1 summarizes the responses regarding LoC.

### 3.3. Views on Aging

**Figure 1:**
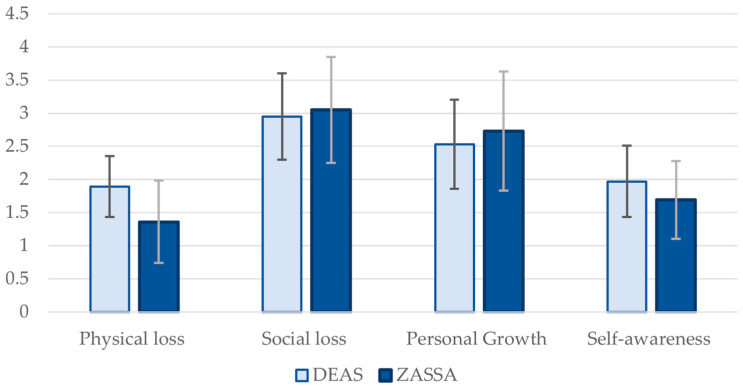
Views on aging domains in the two cohorts (mean and standard deviation).

For the Physical Decline domain, which includes perceptions of loss in health or vitality, the mean score is 1.36 ± 0.62 (median = 1.00, IQR = 0.75). These values indicate a strong agreement among patients that physical decline is a significant aspect of aging. In the Social Loss domain, which includes feelings of no longer being needed by others or decreased respect, the mean score is 3.05 ± 0.80 (median = 3.25, IQR = 1.25). These values suggest a moderate to strong disagreement with the notion that Social Losses are a typical part of aging. A mean score of 2.73 ± 0.90 (median = 2.75, IQR = 1.50) for the Personal Growth domain, reflecting the notion of continuous personal growth, indicates a moderate level of disagreement, suggesting that while some patients believe in the possibility of personal growth as they age, there is significant variability in these perceptions. In the Self-awareness/Gains domain, which includes the development of a better understanding of oneself and one’s limitations, the mean score is 1.69 ± 0.59 (median = 1.50, IQR = 0.75) (Figure 1). These values indicate a strong agreement that Self-awareness/Gains and understanding improve with age.

VoA subscales were not significantly associated with age or HS. Gender-related differences were only observed for Personal Growth, with higher values in male (M = 2.95, SD = 0.80) compared to female patients (M = 2.59, SD = 0.95) (*p* = 0.048).

On the item-level (Supplement Table S2), the older patients perceive aging predominantly in terms of Physical Loss, with 69.9% feeling less resilient, 64.1% less able to compensate for physical limitations, 55.3% less energetic and fit, and 52.4% experiencing health deterioration. Socially, 35.9% disagree with the notion that older people are no longer needed, 58.3% do not get bored more often, 52.4% do not feel less respected, and 53.4% do not feel lonelier. In terms of Personal Growth, 39.8% disagree with the notion of making new plans, 35.9% believe they can still learn new things, 35.9% disagree that they can implement new ideas, and 39.6% do not see their skills expanding. However, Self-Awareness/Gains are recognized, with 38.6% feeling better at handling physical weaknesses, 62.4% having better self-knowledge and awareness of limitations, 71.8% feeling more relaxed, and 52.4% having a clearer idea of what they want as they age.

In summary, these findings highlight that while physical decline is widely acknowledged as an aspect of aging, there is less consensus about Social Losses. The perception that older age leads to being less needed is endorsed by nearly two-thirds of older patients, while the patients are split relatively in half in their perception of boredom, lack of respect, and loneliness. Personal growth is viewed with moderate skepticism, though some variability exists, and there is a strong belief in increased Self-awareness/Gains as people age.

### 3.4. Association Between Locus of Control and Views on Aging

Adjusted associations converged across model families for the Physical domain (Table 2): higher internal LoC was associated with more positive appraisals of physical aging (robust OLS β = 0.133, 95% CI 0.043–0.223, FDR-*p* = 0.035; CLM OR = 3.52, 95% CI 1.31–13.10), whereas higher external LoC related to less positive physical appraisals (β = −0.165, 95% CI −0.285 to −0.045, FDR-*p* = 0.035; OR = 0.274, 95% CI 0.10–0.65). For Social Loss, effects were smaller: the external LoC showed a weak negative association in OLS (β = −0.149, 95% CI −0.314 to 0.016, FDR-*p* = 0.217) and a borderline lower OR in CLM (OR = 0.649, 95% CI 0.418–0.991); internal LoC was not associated. For Personal Growth, internal LoC showed a consistent positive signal (CLM OR = 1.64, 95% CI 1.04–2.72), while the OLS coefficient did not reach significance (β = 0.154, 95% CI −0.036 to 0.345, FDR-*p* = 0.231); external LoC was null. No meaningful associations emerged for Gains. Univariate Spearman correlations were directionally consistent with the modeling: internal LoC correlated positively with phys_pos (r = 0.206, *p* = 0.039, BH-*p* = 0.077) and grow_pos (r = 0.273, *p* = 0.0058, BH-*p* = 0.023), while external LoC correlated negatively with phys_pos (r = −0.317, *p* = 0.0013, BH-*p* = 0.010) and soc_pos (r = −0.238, *p* = 0.0166, BH-*p* = 0.044). Partial Spearman correlations adjusting for age, sex, Barthel, MMSE, GDS, and health satisfaction further supported the pattern for internal LoC with phys_pos (ρ = 0.257, *p* = 0.0176) and grow_pos (ρ = 0.215, *p* = 0.0477).

Overall, the data suggest that a stronger internal control belief is linked to more positive views specifically about physical aging (less perceived Physical Loss) and to a growth-oriented outlook, whereas a stronger external control belief is linked to less positive views on physical (and to a lesser extent social) aging. After correction for multiple testing, the Physical domain shows the most robust pattern; signals in Social Loss (external LoC) and Personal Growth (internal LoC) are weaker and should be interpreted cautiously.

### 3.5. Comparison with the DEAS Cohort

After matching, the two groups—ZASSA and DEAS—showed no significant differences in age, gender distribution, or number of medications, indicating that the matching process effectively balanced these demographic and health-related characteristics (Supplement Table S3). Significant differences emerged in two dimensions of VoA (Figure 1). The older patient group showed lower raw scores on Physical Loss than the matched DEAS group (Supplement Table S3). On the original raw scale (1 = agree to 4 = disagree), lower means indicate stronger agreement with losses, i.e., a more negative appraisal of the physical domain in older patients compared to DEAS. Similarly, raw scores on Self-awareness/Gains were lower in patients than in matched DEAS (Supplement Table S3). On the original scale, lower means indicate stronger agreement with gains, i.e., a more positive appraisal of gains in the patients. No significant differences were observed for Social Loss perceptions or continuous personal growth, as both groups reported similar mean values.

### 3.6. Sensitivity Analyses

To assess the robustness of the findings, additional analyses were conducted by including unmatched DEAS participants. This allowed for a broader comparison between the patients, the matched DEAS sample, and the remaining, unmatched DEAS participants (analyses are provided in the Supplementary Materials in the section 1 “Supplement Sensitivity analyses”). These results highlight that the unmatched DEAS group differs substantially from both the patients and the matched DEAS participants, particularly in age and health status, which are likely to influence their perceptions of aging. Including only the matched DEAS sample in the primary analyses ensured that the comparison focused on individuals with similar demographic and health characteristics, thereby reducing potential confounding effects.

## 4. Discussion

This study analyzed the relationship between VoA and LoC among older patients undergoing early rehabilitative hospital treatment. The results indicate that a majority of patients exhibited a strong internal LoC, perceiving themselves as active agents in their own aging process. At the same time, a predominant perception of aging as a period of physical decline was observed, whereas Social Losses were less frequently reported. Interestingly, individuals with a stronger internal LoC were more likely to perceive aging as a time of personal growth and Self-awareness/Gains, whereas those with external control beliefs tended to associate aging with physical and Social Losses.

The findings align with established theoretical frameworks on aging, particularly Heckhausen and Schulz’s Life-Span Theory of Control (1995) and Brandtstädter and Greve’s Model of the Aging Self (1994). The high prevalence of an internal LoC in the older patients suggests that, even among individuals experiencing functional decline, many retain a sense of agency over their lives. This supports the notion that aging is not solely a process of decline but also an adaptive phase in which individuals engage in secondary control strategies, adjusting their expectations and psychological coping mechanisms to maintain well-being [3,20].

A crucial aspect of the findings concerns the way VoA influences psychological adaptation. The notion that an internal LoC fosters a positive outlook on aging is well-supported in the literature [6,10]. It is well-documented that individuals with an internal LoC are more likely to engage in proactive health behaviors, maintain self-efficacy, and resist age-related stereotypes [1]. Conversely, external control beliefs have been linked to passive adaptation, feelings of helplessness, and disengagement from health-promoting behaviors [13]. The association between external LoC and perceptions of physical and Social Loss in this study reinforces these findings, highlighting how perceived lack of control can contribute to a more negative aging experience or, conversely, how negative aging experiences may lead to a diminished sense of control over one’s life [21]. Individuals with external control beliefs are less able to maintain self-consistency in the face of aging-related changes.

In the comparison with the DEAS dataset, and using the original VoA scaling (1 = agree, 4 = disagree), the older patients showed greater endorsement of Physical Loss (i.e., more perceived physical decline) and greater endorsement of Gains (i.e., more agreement with self-awareness/benefits of aging), whereas Personal Growth was less endorsed than in the DEAS sample. A plausible explanation is that hospitalized patients in early rehabilitation may, due to acute health constraints, hold lower expectations for ongoing personal development, while simultaneously recognizing gains in self-awareness and prioritizing the restoration of autonomy in daily life. These patterns underscore the role of current health status and care context in shaping VoA and suggest that health-related interventions and counseling should explicitly account for such domain-specific variations.

Understanding the relationship between LoC and VoA is crucial for designing interventions that promote healthy aging. Previous theoretical models, such as the lifespan approach to VoA, provide insight into why LoC and VoA are so closely linked: in a recent encompassing article, Kornadt and colleagues combine multiple aging theories to depict the development of VoA across a person’s lifespan. The authors highlight the ever-changing nature of health, social connectedness and roles, as well as aging experiences and attitudes [2]. According to the authors, VoA shape and change across the lifespan as dynamic and complex interactions of biology, psychology and social factors; this multidimensional and ever-changing nature itself poses a challenge to perceived control. Especially in young adulthood, VoA are often associated with loss of control and autonomy, and experimental research shows an immediate effect of perceived control on younger subjective age [22]. Especially in older adults, where changes in health and social roles may occur at any point, feeling like the aging process is controllable rather than fixed or predetermined may lead to more proactive health behavior in favor of better well-being [6,23].

The findings emphasize the importance of reinforcing internal control beliefs in older adults, as these are correlated with greater psychological resilience, adaptive coping strategies, and a more positive perception of aging all of which are related to increased longevity [20,24,25]. Psychological and behavioral interventions that target internal LoC—such as self-efficacy training, goal-setting strategies, and cognitive restructuring of age-related beliefs—could foster more positive VoA and improve overall well-being.

Moreover, the study underscores the importance of addressing age-related stereotypes. Since VoA are shaped by societal and cultural narratives, interventions should also focus on challenging negative stereotypes about aging and fostering environments that support older adults in maintaining autonomy and engagement. Such cultural and environmental factors, fostering resilience and social contact, contribute positively to longevity [24]. Longitudinal studies have shown that positive VoA are linked to better health outcomes and even increased longevity [6]. This is because positive VoA, reflecting a mindset that incorporates growth and development rather than loss of capability and participation, may increase motivation to perform appropriate health behavior even in the face of age-related challenges [26].

Therefore, shifting public discourse towards a more empowered and growth-oriented view of aging has significant public health implications.

Several limitations should be considered when interpreting the results. First, the study’s cross-sectional design precludes causal inferences regarding the relationship between LoC and VoA. Longitudinal research is needed to establish whether changes in LoC precede shifts in aging perceptions or vice versa. Second, the study cohort consisted of hospitalized older patients, which may not be representative of older adults in general. Future studies should compare different subgroups of older adults, including community-dwelling individuals and those in long-term care facilities. Another limitation pertains to the measurement of VoA, which, while based on validated scales, may not fully capture the multidimensional nature of aging perceptions. Future research could incorporate qualitative approaches to gain deeper insights into how older adults conceptualize their own aging experience.

This study contributes to the growing body of research on aging by highlighting the significant interplay between control beliefs and perceptions of aging. The findings reinforce existing theoretical models suggesting that internal control beliefs can promote a more positive, adaptive view of aging, whereas external control beliefs are linked to negative expectations of decline. The study also underscores the importance of health satisfaction in shaping perceptions of aging, suggesting that well-being interventions should focus not only on medical care but also on psychological resilience and self-perception.

From a practical perspective, these findings advocate for the development of empowerment-based interventions aimed at fostering internal control beliefs and challenging negative age stereotypes. Given the well-documented impact of VoA on health outcomes, promoting a growth-oriented perspective on aging should be a priority in geriatric care, rehabilitation, and public health policy.

Ultimately, aging is not merely a biological process but a subjective and socially constructed experience. By enhancing older adults’ sense of agency and reinforcing the idea that personal growth is possible at any stage of life, we can contribute to a more positive, fulfilling aging experience.

## Figures and Tables

**Table 1:** Comprehensive characteristics of the ZASSA cohort (N = 103).

Age (years), mean ± SD		82.38 ± 5.34
Geriatric Depression Scale, mean ± SD		2.86 ± 2.85
Mini-Mental-Status-Examination, mean ± SD		26.97 ± 2.74
Barthel index sum score, mean ± SD		34.85 ± 11.62
VoA—Physical Losses, mean ± SD		1.36 ±0.62
VoA Social Losses, mean ± SD		3.05 ± 0.80
VoA Personal Growth, mean ± SD		2.73 ± 0.90
VoA Gains, mean ± SD		1.69 ± 0.59
LoC Internal, mean ± SD		4.29 ± 0.83
LoC External, mean ± SD		2.44 ± 1.02
Sex, n (%)	Female	63 (61.2)
	Male	40 (38.8)
Marital Status, n (%)	Single	2 (2.0)
	Married	29 (28.4)
	Widowed/Separated	71 (69.6)
Living situation, n (%)	Alone	56 (56.0)
	With Partner/Family	37 (37.0)
	Others	7 (7.0)
LoC = Locus of Control, VOA = Views on Ageing		

**Table 2:** Adjusted associations of internal and external LoC with harmonized VoA domains.

VoA Domain (Higher = More Positive)	Internal LoC—OLS β (95% CI), FDR-*p*	External LoC—OLS β (95% CI), FDR-*p*	Internal LoC—CLM OR (95% CI)	External LoC—CLM OR (95% CI)
Physical Loss	0.133 (0.043–0.223), 0.035	−0.165 (−0.285 to −0.045), 0.035	3.52 (1.31–13.10)	0.274 (0.10–0.65)
Social Loss	0.067 (−0.078–0.213), 0.539	−0.149 (−0.314–0.016), 0.217	1.24 (0.83–1.89)	0.649 (0.418–0.991)
Personal Growth (reversed)	0.154 (−0.036–0.345), 0.231	0.021 (−0.208–0.249), 0.858	1.64 (1.04–2.72)	1.05 (0.67–1.64)
Gains (reversed)	0.054 (−0.093–0.202), 0.539	−0.069 (−0.233–0.095), 0.539	1.25 (0.84–1.85)	0.759 (0.51–1.14)

Notes: VoA harmonization: phys_pos = subjalt_physLoss, soc_pos = subjalt_SocLoss, grow_pos = 5−subjalt_PerGrow, gains_pos = 5−subjalt_Gain. Predictors standardized (β per +1 SD). OLS adjusted for age, sex, Barthel, MMSE, GDS, and health satisfaction; HC3 standard errors; FDR across 8 LoC effects. CLM uses quartiles of each harmonized VoA scale; OR > 1 indicates higher odds of more positive categories; proportional-odds tests were unremarkable.

## Data Availability

The datasets used and/or analyzed during the current study are available from the corresponding author on reasonable request. (The data are not publicly available due to privacy or ethical restrictions).

## Supplementary Materials

The following supporting information can be downloaded at: https://www.mdpi.com/article/10.3390/geriatrics10060148/s1, Table S1: Locus of control; Table S2: Views on aging; Table S3: Comparison between ZASSA and matched DEAS cohort; Table S4: Descriptive statistics for age, gender, views on aging subscales, and medications by group; Supplement Section 1: Sensitivity analyses; Figure S1: VoA—item percentages: physical loss; Figure S2: VoA—item percentages: social loss; Figure S3: VoA—item percentages: personal growth; Figure S4: VoA—item percentages: self-awareness/gains.

## Abbreviations

AIC	Akaike Information Criterion
BH	Benjamini–Hochberg (false discovery rate procedure)
CI	Confidence Interval (e.g., 95% CI)
CLM	Cumulative Link Model (proportional-odds ordinal regression)
DEAS	German Ageing Survey
FDR	False Discovery Rate
GDS	Geriatric Depression Scale
HC3	Heteroskedasticity-Consistent (Type 3) covariance estimator
HS	Health Satisfaction
IE-4	Internal–External Locus of Control short scale (4 items)
LoC	Locus of Control (internal, external)
MMSE	Mini-Mental State Examination
OLS	Ordinary Least Squares
OR	Odds Ratio
SD	Standard Deviation
SE	Standard Error
SMD	Standardized Mean Difference
VIF	Variance Inflation Factor
VoA	Views on Aging
ZASSA	(Name of the hospital cohort; acute geriatric rehabilitation sample)
