# Supplementary material for: Perceptions of Aging and Control Beliefs: A Study on Older Patients’ Views of Aging
Source: Geriatrics (Basel). 2025 Nov 10;10(6):148. doi: 10.3390/geriatrics10060148 (PMC12641807; doi:10.3390/geriatrics10060148)
Supplement: Supplementary file 1 [file geriatrics-10-00148-s001.zip › geriatrics-3923101-supplementary.pdf]

geriatrics

MDPI

## Supplement

**Supplement Table S1. Locus of control.**

| | | | n | % |
|---|---|---|---|---|
| Internal | I'm my own boss. | Does not apply at all | 2 | 1.9 |
| | | Applies a bit | 10 | 9.7 |
| | | Applies somewhat | 6 | 5.8 |
| | | Applies mostly | 12 | 11.7 |
| | | Applies completely | 73 | 70.9 |
| | If I work hard, I will succeed. | Does not apply at all | 0 | 0.0 |
| | | Applies a bit | 10 | 9.7 |
| | | Applies somewhat | 12 | 11.7 |
| | | Applies mostly | 30 | 29.1 |
| | | Applies completely | 51 | 49.5 |
| External | Whether at work or in my private life, what I do is mainly determined by others. | Does not apply at all | 70 | 68.0 |
| | | Applies a bit | 12 | 11.7 |
| | | Applies somewhat | 1 | 1.0 |
| | | Applies mostly | 12 | 11.7 |
| | | Applies completely | 8 | 7.8 |
| | Fate often gets in the way of my plans. | Does not apply at all | 23 | 22.3 |
| | | Applies a bit | 21 | 20.4 |
| | | Applies somewhat | 7 | 6.8 |
| | | Applies mostly | 32 | 31.1 |
| | | Applies completely | 20 | 19.4 |
| | | | **M** | **SD** |
| | Internal locus of control, mean | | 4.29 | 0.83 |
| | External locus of control, mean | | 2.44 | 1.02 |

* German Internal–External Locus of Control Short Scale–4 (IE-4) scale (Kovaleva et al., 2012): https://zis.gesis.org/skala/Nießen-Schmidt-Groskurth-Rammstedt-Lechner-Internal–External-Locus-of-Control-Short-Scale–4-(IE-4).

**Supplement Table S2. Views on aging.**

| For most people, getting older means ... | | n | % |
|---|---|---|---|
| **Physical Loss** | | | |
| 1. that you aren't able to withstand as much as you used to | Strongly agree | 72 | 69.9 |
| | Agree | 22 | 21.4 |
| | Disagree | 7 | 6.8 |
| | Strongly disagree | 2 | 1.9 |
| 2. that you are less able to compensate for physical limitations | Strongly agree | 66 | 64.1 |
| | Agree | 24 | 23.3 |
| | Disagree | 10 | 9.7 |
| | Strongly disagree | 3 | 2.9 |
| 3. that you are less energetic and fit | Strongly agree | 57 | 55.3 |
| | Agree | 31 | 30.1 |
| | Disagree | 11 | 10.7 |
| | Strongly disagree | 4 | 3.9 |
| 4. that your health gets worse | Strongly agree | 54 | 52.4 |
| | Agree | 26 | 25.2 |
| | Disagree | 18 | 17.5 |

| | | | |
|---|---|---|---|
| | Strongly disagree | 5 | 4.9 |
| **Social Loss** | | | |
| 5. that you aren't really needed anymore | Strongly agree | 21 | 20.4 |
| | Agree | 24 | 23.3 |
| | Disagree | 21 | 20.4 |
| | Strongly disagree | 37 | 35.9 |
| 6. that you get bored more and more often | Strongly agree | 7 | 6.8 |
| | Agree | 17 | 16.5 |
| | Disagree | 19 | 18.4 |
| | Strongly disagree | 60 | 58.3 |
| 7. that people treat you with less respect | Strongly agree | 6 | 5.8 |
| | Agree | 20 | 19.4 |
| | Disagree | 23 | 22.3 |
| | Strongly disagree | 54 | 52.4 |
| 8. that you feel lonely more often | Strongly agree | 18 | 17.5 |
| | Agree | 21 | 20.4 |
| | Disagree | 9 | 8.7 |
| | Strongly disagree | 55 | 53.4 |
| **Personal Growth** | | | |
| 9. that you keep making plans | Strongly agree | 25 | 24.3 |
| | Agree | 14 | 13.6 |
| | Disagree | 23 | 22.3 |
| | Strongly disagree | 41 | 39.8 |
| 10. that you're still able to learn new things | Strongly agree | 30 | 29.1 |
| | Agree | 37 | 35.9 |
| | Disagree | 11 | 10.7 |
| | Strongly disagree | 25 | 24.3 |
| 11. that you can still put your ideas into practice | Strongly agree | 12 | 11.7 |
| | Agree | 22 | 21.4 |
| | Disagree | 32 | 31.1 |
| | Strongly disagree | 37 | 35.9 |
| 12. that you can expand your skills and abilities | Strongly agree | 11 | 10.9 |
| | Agree | 22 | 21.8 |
| | Disagree | 28 | 27.7 |
| | Strongly disagree | 40 | 39.6 |
| **Self-awareness / Gains** | | | |
| 13. that you are better able to deal with physical weaknesses | Strongly agree | 35 | 34.7 |
| | Agree | 39 | 38.6 |
| | Disagree | 20 | 19.8 |
| | Strongly disagree | 7 | 6.9 |
| 14. that you know yourself and your limitations better | Strongly agree | 63 | 62.4 |
| | Agree | 22 | 21.8 |
| | Disagree | 8 | 7.9 |
| | Strongly disagree | 8 | 7.9 |
| 15. that you are more relaxed about a lot of things | Strongly agree | 74 | 71.8 |
| | Agree | 12 | 11.7 |
| | Disagree | 13 | 12.6 |
| | Strongly disagree | 4 | 3.9 |
| 16. that you have a better idea of what you want | Strongly agree | 54 | 52.4 |
| | Agree | 31 | 30.1 |

| | | |
|---|---|---|
| Disagree | 14 | 13.6 |
| Strongly disagree | 4 | 3.9 |

**Supplement Table S3. Comparison between ZASSA and matched DEAS cohort.**

| Variable | | Matched DEAS | | ZASSA | |
|---|---|---|---|---|---|
| | | N | % | N | % |
| Gender | female | $62_a$ | 62.0 | $63_a$ | 61.2 |
| | male | $38_a$ | 38.0 | $40_a$ | 38.8 |
| | | M | SD | M | SD |
| Age | | $82{,}25_a$ | 5.54 | $82.38_a$ | 5.34 |
| Physical loss | | $1.89_a$ | 0.46 | $1.36_b$ | 0.62 |
| Social Loss | | $2.95_a$ | 0.65 | $3.05_a$ | 0.80 |
| Personal Growth | | $2.53_a$ | 0.67 | $2.73_a$ | 0.90 |
| Self-awareness / Gains | | $1.97_a$ | 0.54 | $1.69_b$ | 0.59 |
| Number of medications | | $8.40_a$ | 3.99 | $8.48_a$ | 4.06 |

* Note: Raw values are provided for Views on aging domains (lower values = more agreement). Values in the same row where the subscript is not identical differ strongly at $p < 0.05$.

## Supplement Sensitivity analyses

To assess the robustness of the findings, additional analyses were conducted by including unmatched DEAS participants. This allowed for a broader comparison between the ZASSA cohort, the matched DEAS sample, and the remaining, unmatched DEAS participants. These sensitivity analyses are provided in the supplement.

These sensitivity analyses revealed substantial differences between the unmatched DEAS group and the other two groups in key demographic and views on aging (VoA) variables (**Supplement Table S4**).

Unmatched DEAS participants were significantly younger, with a mean age of 68.18 years (SD = 10.61), compared to the ZASSA group (M = 82.38, SD = 5.34) and the matched DEAS sample (M = 82.25, SD = 5.54, $p < 0.05$). The gender distribution in the unmatched DEAS group was also more balanced, with 50.6% female and 49.4% male participants, whereas the ZASSA and matched DEAS groups exhibited a higher proportion of female participants (61.2% and 62.0%, respectively).

The unmatched DEAS group demonstrated significantly different VoA patterns compared to both the ZASSA and the matched DEAS samples. On the original scale (1=agree–4=disagree), higher means reflect less agreement with losses. Thus, the unmatched DEAS group (M = 2.27) actually shows less perceived Physical Loss than ZASSA (M = 1.36), with matched DEAS in between (M = 1.89). A similar logic applies to Social Loss (higher = less loss), where unmatched DEAS participants had the highest scores (M = 3.20), indicating fewer perceptions of age-related social decline, while both the ZASSA and matched DEAS groups reported significantly lower values.

Regarding Personal Growth, unmatched DEAS participants reported the lowest raw scores (M = 2.07, SD = 0.55), indicating greater endorsement of aging as a period of continuous development. The ZASSA group exhibited the highest scores (M = 2.73, SD = 0.90), indicating less endorsement, with the matched DEAS group in between (M = 2.53, SD = 0.67, $p < 0.05$). However, perceptions of Self-awareness / Gains in aging were similar between the unmatched DEAS and matched DEAS groups (M = 1.94, SD = 0.45 vs. M = 1.97, SD = 0.54, n.s.), while the ZASSA group reported significantly lower scores (M = 1.69, SD = 0.59, $p < 0.05$).

A notable difference was also observed in the number of medications taken. The unmatched DEAS group had a markedly lower mean medication count (M = 2.54, SD =

2.64), compared to the ZASSA group (M = 8.48, SD = 4.06) and the matched DEAS group (M = 8.40, SD = 3.99, $p < 0.05$).

**Supplement Table S4. Descriptive statistics for age, gender, views on aging subscales, and medications by group.**

| Variable | DEAS (not matched) (N = 5,302) | ZASSA (N = 103) | DEAS (matched) (N = 100) |
|---|---|---|---|
| Age | 68.18 (10.61) $_a$ | 82.38 (5.34) $_b$ | 82.25 (5.54) $_b$ |
| Female, n (%) | 2,684 (50.6%) | 63 (61.2%) | 62 (62.0%) |
| Male, n (%) | 2,618 (49.4%) | 40 (38.8%) | 38 (38.0%) |
| Physical Loss | 2.27 (0.55) $_a$ | 1.36 (0.62) $_b$ | 1.89 (0.46) $_c$ |
| Social Loss | 3.20 (0.55) $_a$ | 3.05 (0.80) $_b$ | 2.95 (0.65) $_b$ |
| Personal growth | 2.07 (0.55) $_a$ | 2.73 (0.90) $_b$ | 2.53 (0.67) $_c$ |
| Self-awareness / Gains | 1.94 (0.45) $_a$ | 1.69 (0.59) $_b$ | 1.97 (0.54) $_a$ |
| Number of medications | 2.54 (2.64) $_a$ | 8.48 (4.06) $_b$ | 8.40 (3.99) $_b$ |

* Variables (Gender breakdown). Values in the same row where the subscript is not identical differ strongly at $p< 0.05$.

**Supplement Figure S1** VoA – item percentages: physical loss.

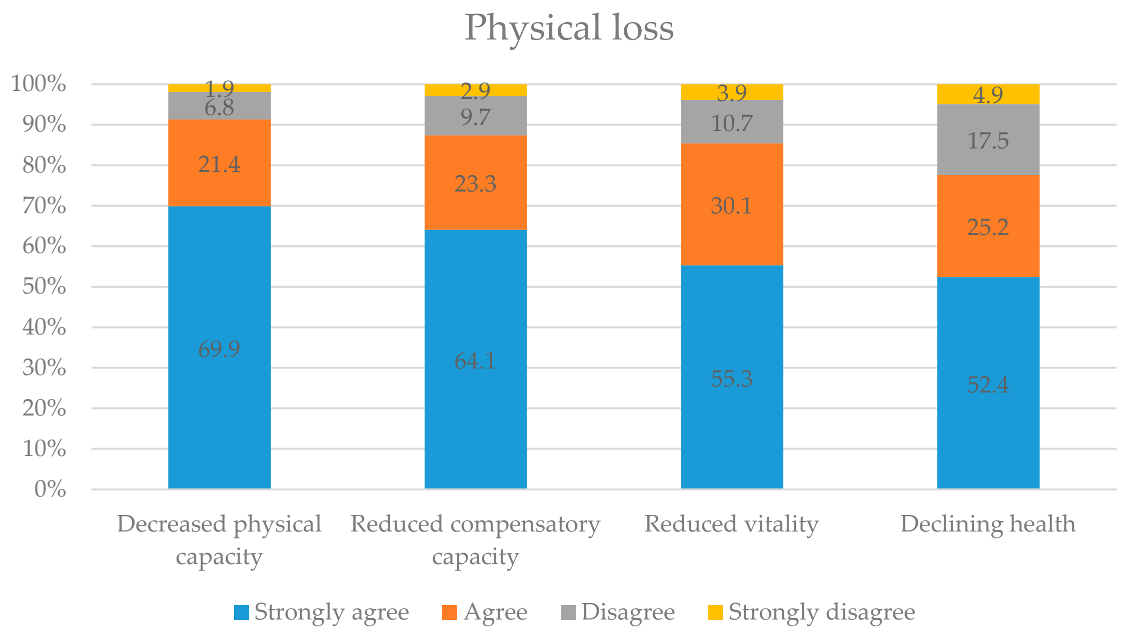


**Supplement Figure S2** VoA – item percentages: social loss.

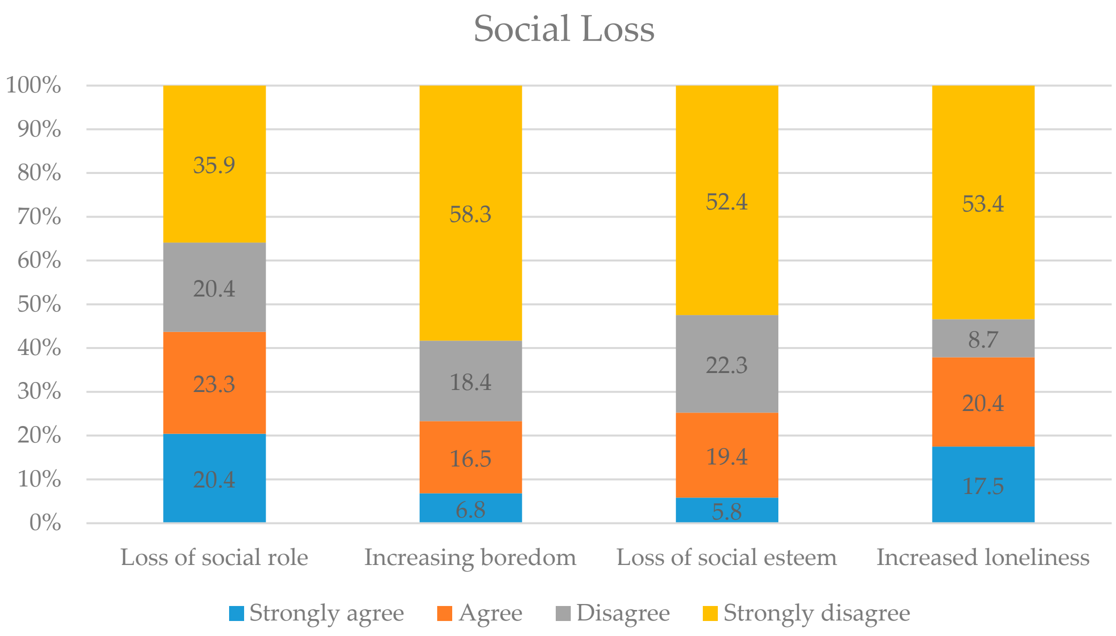


**Supplement Figure S3** VoA – item percentages: personal growth.

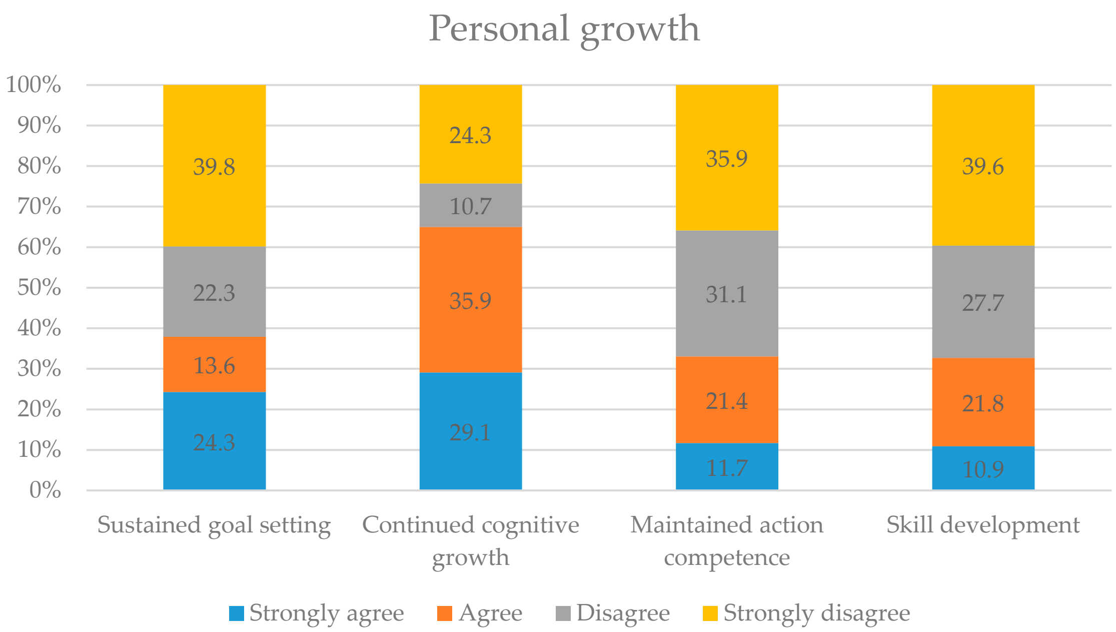


**Supplement Figure S4** VoA – item percentages: self-awareness/gains.

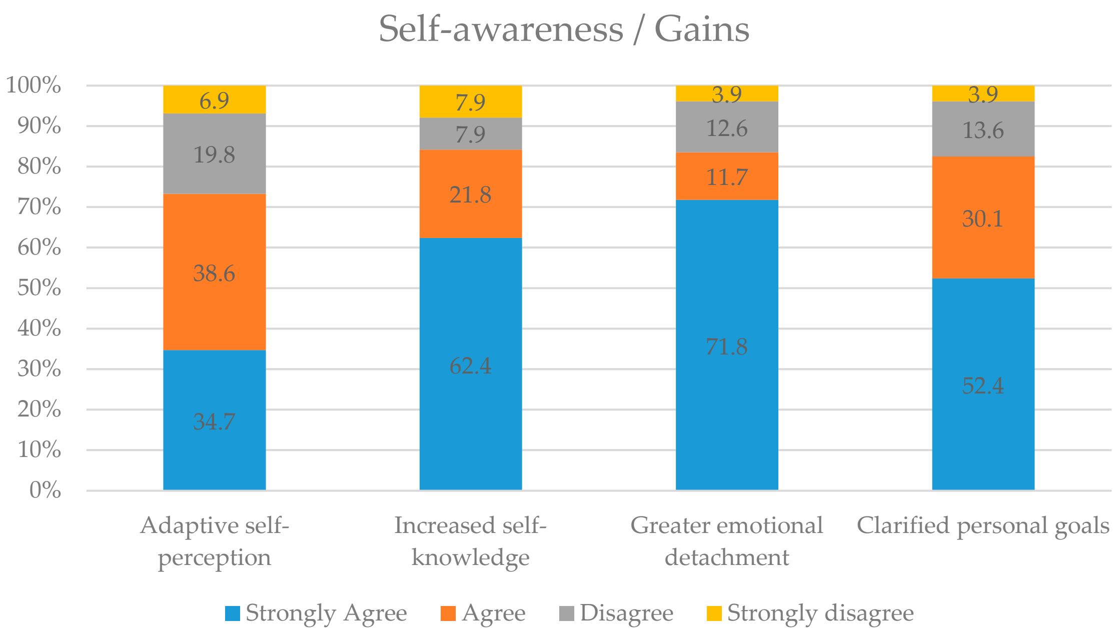
Self-awareness / Gains
100%
90%
80%
70%
60%
50%
40%
30%
20%
10%
0%
6.9
19.8
38.6
34.7
7.9
7.9
21.8
62.4
3.9
12.6
11.7
71.8
3.9
13.6
30.1
52.4
Adaptive self-perception
Increased self-knowledge
Greater emotional detachment
Clarified personal goals
Strongly Agree
Agree
Disagree
Strongly disagree
